# Is re-Rex shunt a better choice for patients with failed Rex shunt?

**DOI:** 10.3389/fped.2023.1135059

**Published:** 2023-06-26

**Authors:** Zhe Wen, Jieqin Wang, Chao Yang, Tao Liu, Qifeng Liang, Jiankun Liang, Yu Ning, Fuyu You, Xiaoling Bai, Miao Hong

**Affiliations:** ^1^ Department of Pediatric Surgery, Guangzhou Women and Children's Medical Center, Guangdong Provincial Clinical Research Center for Child Health, Guangzhou Medical University, Guangzhou, China; ^2^ Clinical Data Center, Guangzhou Women and Children's Medical Center, Guangdong Provincial Clinical Research Center for Child Health, Institute of Pediatrics, Guangzhou Medical University, Guangzhou, China

**Keywords:** extrahepatic portal venous obstruction, portal hypertension, rex shunt, warren shunt, reoperation

## Abstract

**Purpose:**

To review our single-center surgical outcomes of redo operations after failed Rex shunt procedures.

**Methods:**

From September 2017 to October 2021, a total of 20 patients (11 males, 9 females; median age: 8.6 years) with Rex shunt occlusions were admitted to our hospital. Two of these patients were previously operated on in our hospital, and the remaining 18 were from other centers. All patients underwent repeat operations after detailed preoperative evaluations.

**Results:**

Preoperative wedged hepatic vein portography (WHVP) was conducted for 18 patients. Thirteen patients exhibited well-developed Rex recessus and intrahepatic portal vein during WHPV examination, consistent with the intraoperative exploration results. Fifteen patients (75%, 15/20) underwent redo-Rex shunt, four underwent Warren shunt and one underwent devascularization surgery. During the redo-Rex shunt operations, the left internal jugular veins (IJV) were used as bypass grafts in 11 patients; the intra-abdominal veins were used in 4 patients. The patients were followed up for 12–59 months (mean, 24.8 months). After redo Rex shunts, the grafts were patent in 14 patients (93.3%, 14/15), but 1 graft had thrombosis (6.7%, 1/15). Three patients suffered from postoperative anastomotic stenosis, and all of the stenosis was relieved with balloon dilatations. After re-Rex shunts, esophageal varices and spleen size were substantially reduced, and the platelet count significantly increased. Postoperative graft thrombosis was found in 1 patient after Warren shunt (1/4, 25%), and there was no graft stenosis. Compared with Warren surgery, patients who underwent re-Rex shunt had a significantly higher rate of platelet increase.

**Conclusions:**

Redo-rex shunts can be finished in most patients with failed Rex shunts. Re-Rex shunt is a preferred surgical choice after a failed Rex shunt when a good bypass graft is available, and the surgical success rate can reach more than 90%. A suitable bypass graft is essential for a successful redo Rex shunt. Preoperative WHVP is recommended for the design of a redo surgical plan preoperatively.

## 1. Introduction

Rex shunt has been the preferred surgical treatment for children with extrahepatic portal venous obstruction (EHPVO) (1). However, postoperative bypass graft thrombosis is the leading cause of surgical failure. Reports of further surgical management and the efficacy of these surgical management strategies are rare in the literature. At present, we have successfully finished about 150 Rex shunt procedures, including some repeat Rex shunt procedures. Here, we analyzed our single-center redo-operation outcomes in patients with previously failed Rex shunts.

## 2. Methods

### 2.1. Study design and participants

Between September 2017 and October 2021, a total of 20 children (11 boys, 9 girls; median age 8.6 years; interquartile range: 2 to 17 years) with bypass graft occlusion after Rex shunt were admitted to our hospital. The detailed patient data are shown in Table 1. All of these patients had a previous history of the Rex shunt procedure and developed recurrent gastrointestinal hemorrhage due to bypass graft thrombosis. Bleeding started 2 weeks to 6 years (median duration: 4 months) after the previous operations. Two of the 20 patients were previously operated on in our hospital, and the remaining 18 underwent Rex shunts in other hospitals. A total of 24 Rex shunts were performed for the 20 patients; 17 patients underwent a single operation, 2 had two attempts, and one had three attempts. Some patients received other surgeries for portal hypertension, including total splenectomy, partial splenectomy, ligation of the splenic artery or Warren shunt (Table 1).

**Table 1:** Patient data.

Patients	Gender	Age at first surgery (y)	Age at redo surgery (y)	Times of previous Rex shunt	Previous bypass	Other PHT surgery	Rex recessus	Redo operation	Bypass conduit	Follow up (m)	pre/post operative gastrascopy	pre/post operative PLT	pre/post operative splenic length/height	Postoperative rebleeding	Vascular complication/treatment
1	M	3	5	1	IJV	No	Patent	Rex	CV	59	3/2	83/111	0.113/0.112	Yes	Stenosis/dilatation
2	M	5	6	1	IMV	No	Atretic	Warren	NA	44	3/2	95/71	0.112/0.110	No	None
3	F	5	8	2	CV/SV	Partial splenectomy	Atretic	Devascularization	NA	32	NA	434/460	NA	No	None
4	M	10	13	1	IMV	No	Patent	Rex	IJV	32	3/1	82/138	0.111/0.092	No	None
5	M	11	11	1	ILV	No	Atretic	Warren	NA	45	NA	76/62	NA	No	None
6	F	7	7	1	CV	No	Small	Warren	NA	20	NA	75/97	0.102/0.092	Yes	Thrombosis
7	F	9	12	1	CV	No	Patent	Rex	IMV	13	3/1	117/165	0.099/0.077	No	None
8	F	6	7	1	CV	No	Patent	Rex	IJV	12	3/0	85/151	0.102/0.087	No	None
9	M	4	10	1	SV	Partial splenectomy	Patent	Rex	IJV	32	3/0	175/650	NA	Yes	None
10	M	1	3	1	CV	No	Atretic	Warren	NA	16	2/2	183/216	0.083/0.070	No	None
11	F	2	3	1	CV	No	Patent	Rex	IJV	23	3/0	58/173	0.113/0.074	No	None
12	M	6	9	2	CV/IMV	No	Patent	Rex	IJV	16	3/2	32/62	0.111/0.084	No	Stenosis/dilatation
13	F	4	5	1	CV	Splenectomy	Patent	Rex	IJV	13	3/1	685/500	NA	No	None
14	M	5	15	3	CV/SV/IMV	Splenectomy	Patent	Rex	IJV	28	3/0	639/514	NA	No	None
15	M	6	9	1	RGEV	Warren	Patent	Rex	IMV + CV	26	NA	61/59	0.117/0.113	Yes	Thrombosis
16	M	10	17	1	IJV	Warren	Patent	Rex	SV + PV	25	3/2	48/76	0.124/0.127	No	None
17	F	1	2	1	CV	No	Patent	Rex	IJV	18	3/2	145/131	0.116/0.129	No	Stenosis/dilatation
18	M	4	5	1	IMV	No	Patent	Rex	IJV	14	3/0	84/151	0.129/0.128	No	None
19	F	3	11	1	SV	No	Patent	Rex	IJV	13	NA	38/102	0.138/0.143	No	None
20	F	7	13	1	CV	Warren	Patent	Rex	IJV	15	3/1	83/137	0.096/0.083	No	None

IJV, internal jugular vein; CV, gastric coronary vein; IMV, inferior mesenteric vein; PBV, enlarged pancreatic branch vein; RGEV, right gastroepiploic vein; SV, splenic vein; ILV, ileal vein.

The previous procedures utilized different autologous vein grafts, including two internal jugular veins (IJV, 2), eleven gastric coronary veins (CV, 11), five inferior mesenteric veins (IMV, 5), one right gastroepiploic vein (RGEV, 1), four splenic veins (SV, 4), and one ileal vein (ILV, 1).

### 2.2. Diagnosis and preoperative evaluation

Preoperative abdominal ultrasound and contrast CT examinations confirmed the diagnosis of cavernous transformation of the portal vein (CTPV) and showed complete occlusion of the bypass grafts in all of these patients. Blood tests showed normal liver function and nearly normal coagulation. To assess intrahepatic portal vein conditions, preoperative interventional wedged hepatic vein portographies (WHVPs) were performed on 18 of the patients. The bypass vessels were occluded in all the patients, so interventional recanalization could not be performed.

### 2.3. Surgical procedure

The upper abdominal incisions were reopened according to the original incision, which was either transverse or median longitudinal. After adhesiolysis, the Rex recessus and the previous bypass graft were subsequently exposed. The Rex shunt procedure, as previously reported (2), was preferred if the left portal vein was patent. Otherwise, a Warren shunt procedure was performed. If neither the re-Rex shunt procedure nor the Warren shunt procedure was feasible, a devascularization surgery was performed. Before and after vascular anastomosis, the portal pressure was measured through branches of the superior mesenteric vein. The IJV was the preferred graft in the redo Rex shunt. When the IJV was not available, intra-abdominal vessels such as the CV and IMV were used as bypass grafts.

Intravenous heparin was given for 3 days postoperatively, followed by low-molecular-weight heparin (FRAXIPARINE) for another 3 days. The regimen was then switched to oral dipyridamole and aspirin or warfarin for six months.

### 2.4. Follow-up

All patients were followed up at 1, 3, 6 and 12 months and each year postoperatively afterward. At each visit, complete blood tests and liver function were assessed. Abdominal ultrasonography was carried out to assess the bypass graft diameter, flow velocity and spleen size. To eliminate the effect of the increases in spleen length with age, the ratio of spleen length to height was compared before and after surgery. When necessary, CT scanning was also conducted. To assess the recovery of esophageal and gastric varices, gastroscopy was routinely performed 12 months after surgery. The follow-up period ranged from 12 to 59 months (mean, 24.8 months).

### 2.5. Diagnosis of vascular complications

Postoperative anastomotic stenosis was suspected if ultrasound and CT showed that the diameter of vascular anastomosis was less than 3 mm, the flow rate was more than 3 times that of the bypass vessel, and the patient had corresponding symptoms, such as an enlarging spleen, a decreased platelet count, and recurrence of variceal bleeding (3). Bypass graft occlusion was diagnosed if the bypass grafts could not be detected on ultrasound and CT.

### 2.6. Statistical analysis

The data were analyzed by ANOVA using the SPSS 13.0 (SPSS Inc., IL, USA) package. The results were considered statistically significant when *p* was < 0.05.

## 3. Results

### 3.1. Preoperative assessment of the Rex recessus and intrahepatic portal vein

**Figure 1:**
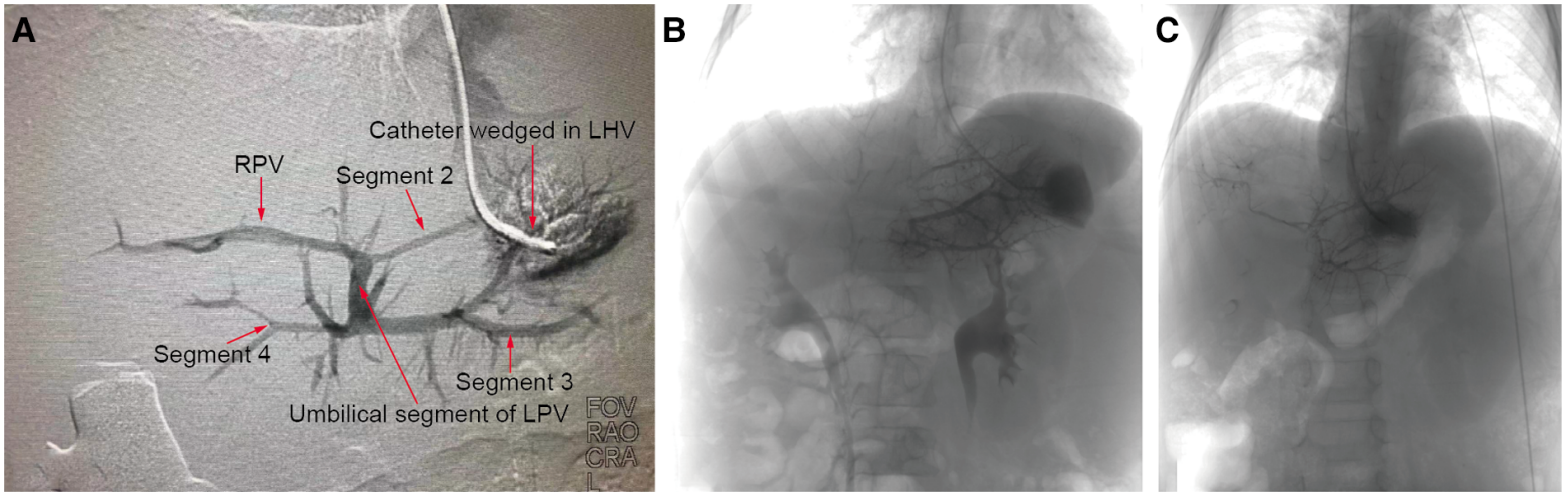
WHVP. Preoperative interventional wedged hepatic vein portography (WHVP) was performed, with a well-developed intrahepatic portal vein in patient 13 (**A**), an invisible Rex recessus in patient 10 (**B**) and a small diameter intrahepatic portal vein in patient 6 (**C**).

To assess the development of the rex recessus and the intrahepatic portal vein, preoperative wedged hepatic vein portography (WHVP) was performed on 18 out of the 20 patients. The WHVP results showed a visible rex recessus and a well-developed intrahepatic portal vein in 13 patients. One patient was found to have a narrow intrahepatic portal vein (patient #6). The Rex recessus was not visible in the other 4 patients (Figure 1).

### 3.2. Reoperative methods

**Figure 2:**
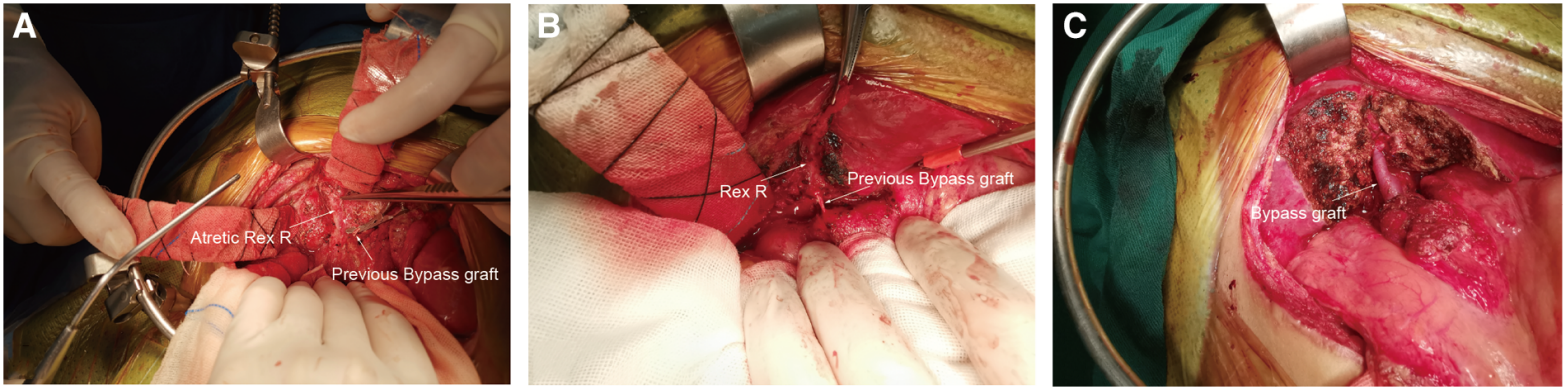
Operative pictures. Rex R: Rex recessus. (**A**), Rex R and the sagittal part of the portal vein were atretic (patient 3). (**B**), Rex recessus and sagittal part of the portal vein were explored to be patent (patient 12). (**C**), The Rex shunt procedure was completed (patient 12).

All 20 patients underwent reoperations. The intraoperative findings were consistent with the preoperative WHVP findings. The Rex recessus was found to be patent in 15 patients, atretic in 4 patients and narrow (only 1.5 mm in diameter) in 1 patient. Ultimately, 15 patients underwent redo Rex shunt surgery, and 4 patients had Warren shunts. Patient #3 with an atretic Rex recessus had a previous Rex shunt with the splenic vein as the bypass graft, thus precluding a redo-Rex shunt and Warren shunt. He finally underwent devascularization surgery (Figure 2).

### 3.3. Bypass graft selection for re-Rex shunt

The left IJV was used as a bypass graft in 11 of the 15 re-Rex shunt patients, and the intraabdominal vessels were utilized in 4 patients. In patient #1, the IJV was already sacrificed in the previous Rex shunt procedure, and the CV was used as the graft in the second surgery. In patient 7, CV was used as a bypass graft in the initial rex shunt, while IMV was used in the redo shunt, with a diameter of 5 mm and a sufficiently long length. Patient #15, who had previous Warren and Rex shunts (using RGEV as the bypass graft), had a dysplastic left IJV, so the IMV was joined with the dilated CV as the bypass graft with eventual diameters of 3 mm–4 mm. Patient #16 had a Warren shunt followed by a Rex shunt with the IJV. At the third operation in our hospital, we found that 5 cm of the distal splenic vein was still patent. We thus harvested the splenic vein and its dilated pancreatic tributary, tailoring a 6 cm bypass graft. A re-Rex shunt was accomplished by an end-to-end anastomosis between the bypass graft and portal vein trunk above the pancreas.

### 3.4. Pathological findings

Liver biopsies from all of the patients demonstrated normal hepatic structures, with no signs of either hepatic cirrhosis or fibrosis.

### 3.5. Bypass graft patency

**Figure 3:**
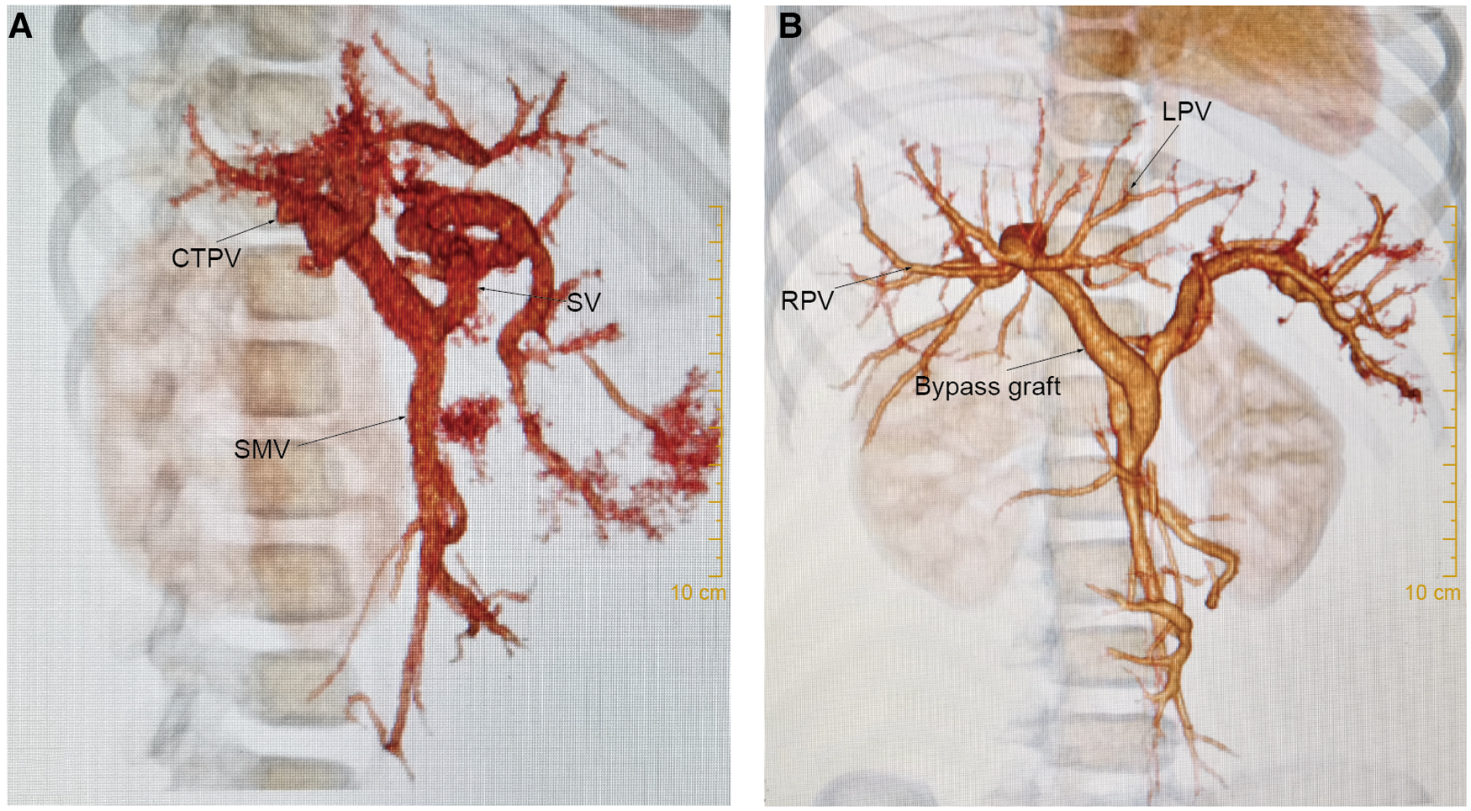
Ct scan. For case 8, (**A**): CT scan showed cavernous transformation of the portal vein before re-Rex shunt. (**B**): The bypass graft was patent 1 year after the re-Rex shunt.

Ultrasonography and CT detected vascular thrombosis after re-Rex in 1 patient (6.7%, 1/15) and anastomotic stenosis in 3 patients (20%, 3/15). All the other 14 patients had patent bypass grafts without vascular complications (see Figure 3). All 3 stenoses were at the anastomotic site at the hepatic end of the bypass vessels. The graft thrombosis rate after Warren shunt was 25% (1/4), with no stenosis observed. Vascular thrombosis was found in patient 15 with a re-Rex shunt and patient 6 with a Warren shunt 1 year after surgery. Anastomotic stenosis was found in 3 patients 1 month to 6 months after redo-Rex surgery, and interventional vascular dilation was performed 1 month to 2 years postoperatively. The anastomotic site diameter increased from 2.0 mm–2.2 mm to 4.3 mm–4.6 mm after dilation, and there was remission of the portal hypertension symptoms associated with hematochezia, splenomegaly and hypersplenism (Table 1).

### 3.6. Postoperative gastrointestinal rebleeding

Postoperative hematochezia occurred in 4 of the 20 patients, 3 of whom had vascular complications of anastomotic stenosis or thrombosis. After redo-Rex surgery, patient 9 suffered repeated bleeding. Although gastroscopy suggested the disappearance of varices, erosive gastritis localized in the gastric fundus were observed. The second surgical exploration revealed that the pressure of the short gastric vessels was significantly higher than that of the mesenteric area, indicating that the local high pressure was caused by poor splenic vein returns through the short gastric vessels, which induced gastric fundus lesions. The hemorrhage disappeared after splenectomy (Table 1).

### 3.7. Varicosity

**Figure 4:**
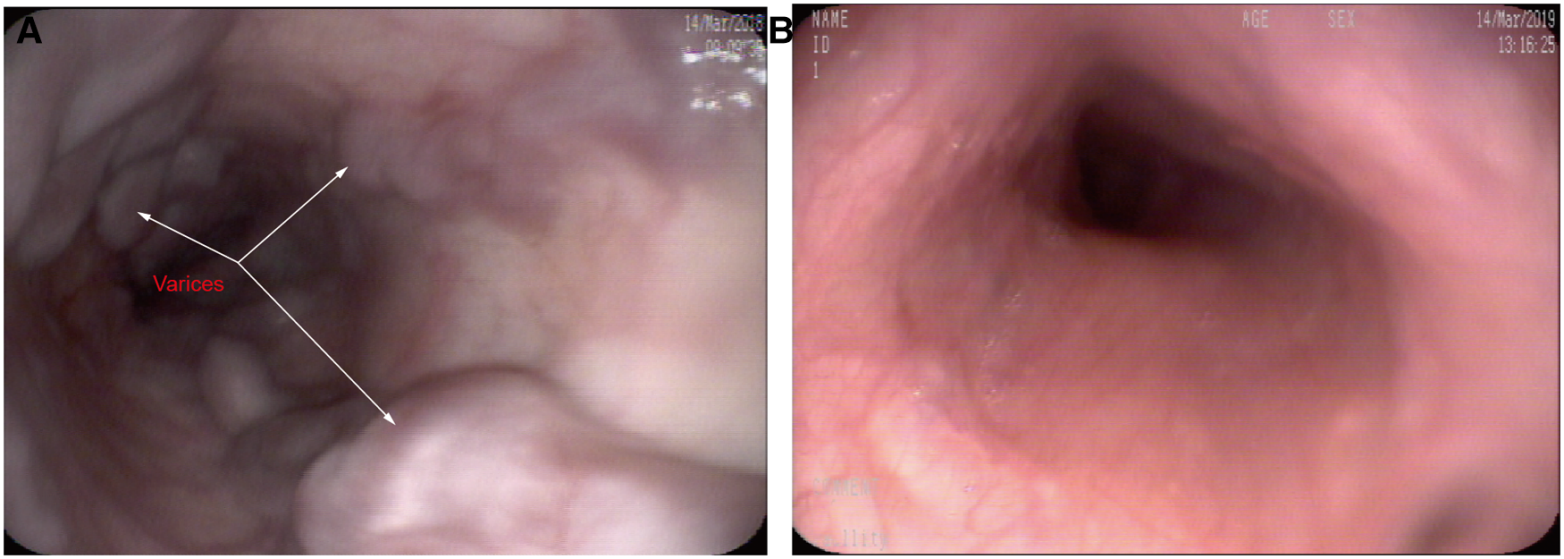
Gastroscopy showing severe esophageal varices before re-Rex shunt (**A**), and the varices had completely disappeared after re-Rex shunt (**B**).

Gastroscopy was performed in 15 patients 1 year after surgery (the patient with bypass thrombosis was excluded, and for patients with anastomotic stenosis, gastroscopy was performed after interventional balloon dilation). All 15 patients had severe esophagogastric varices (grade 2–3) before surgery, which were significantly relieved after surgery. At the 1-year follow-up visit, varices decreased to the grade 0 level and to the 1 level in 5 (33.3%, 5/15) and 4 (26.7%, 4/15) patients, respectively, all of whom were in the re-Rex shunt group. Varices decreased to the grade 2 level in 5 patients (33.3%, 5/15) but were maintained at the same preoperative level in 1 patient (6.7%, 1/15) (See Figure 4).

### 3.8. Splenomegaly and hypersplenism

Of the 20 redo patients, 2 had previous splenectomy, 2 had partial splenectomy, and 3 had Warren surgeries. The splenic length/height ratios and the platelet counts improved in most patients after redo surgery, especially in the re-Rex group (patients with bypass thrombosis or splenectomy or missing data were excluded).

After stratification by the reoperation method, we found that patients who underwent re-Rex shunt had a higher rate of relief of varices as well as platelet increase than that of the Warren group, and there was a statistically significant difference in the rate of platelet increase between the two groups (*p* = 0.03). Further subgrouping according to the bypass conduit selection, both a higher rate of platelet increase and patency rate were observed in patients with IJV bypass, although the differences did not reach statistical significance (Tables 2,3).

**Table 2:** Comparison between Rex shunt and warren shunt.

Redo operation	Relief of varices	Platelet increase		Relief of splenomegaly
Rex shunt	13/13, 100%		11/11, 100%	7/11, 72.7%
Warren shunt	1/2, 50%		1/3, 33.3%	2/2, 100%
*P*	0.13		0.03*	0.54

Analysis of variance (ANOVA).

* Statistically significant.

**Table 3:** Comparison between different kinds of bypass grafts.

Bypass grafts	Relief of varices	Platelet increase	Relief of splenomegaly	Patency rate
IJV	10/10,100%	8/8,100%	5/8,62.5%	11/11,100%
Others	3/3,100%	3/4,75%	3/4,75%	3/4,75%
*P*	–	0.33	1	0.27

Analysis of variance (ANOVA).

## 4. Discussion

Since its introduction by Jean de Ville de Goyet in 1992(4), the Rex shunt procedure has been widely accepted as the preferred surgical option for EHPVO (1). Unlike PSS, Rex shunt is a radical procedure for treating EHPVO with satisfactory results, which not only tackles severe portal hypertension (PH) complications such as variceal hemorrhage and hypersplenism but also rectifies PH-associated problems by restoring portal vein inflow, such as developmental disorders (5), coagulopathy (6) and hepatic encephalopathy (7). However, previous studies have reported an incidence of shunt thrombosis between 4% and 25% in these patients (8–10), which results in surgical failure. Currently, there is little literature on surgical management after rex shunt failure, so it is necessary to further discuss this issue.

Because the Rex shunt is a radical operation for the treatment of prehepatic portal hypertension, it has been regarded as the first treatment choice for EHPVO patients (1). Although conservative treatment with endoscopic ligation/sclerosis can be considered to relieve portal hypertensive symptoms in patients in whom the Rex shunt procedure failed, a repeat Rex surgery should still be considered first, when vascular conditions permit. However, the thromboses within the bypass vessels may spread to the liver and cause Rex recessus atresia, and a previous surgical operation may damage the integrity of the Rex recessus. Thus, the opportunity for Rex surgery may be lost. Among the 20 patients in our study, 15 (75%) patients still had a patent rex recessus, and the re-Rex shunt could be finished. However, 5 (25%) patients had an atretic or dysplasia Rex recessus during surgical exploration, and there was no chance of re-Rex surgery. It is interesting that the SMV and SV remained patent in all 20 patients and were not affected by bypass thrombosis. In a study including 12 patients with failed rex shunts by Zhang et al. (11), rex reccessus stenosis was found in 4 patients, and the re-Rex shunt feasibility rate was 66% (8/12), which is similar to the results of our research. These results indicate that in most patients with failed rex shunts, the rex recessus remains intact or is slightly damaged, which makes redo rex surgery feasible.

According to previous studies, the success rate of the re-Rex shunt is significantly reduced compared with that of the primary Rex shunt. Bhat et al. (12) reported 9 patients undergoing the re-Rex procedure and showed a bypass thrombotic rate of 66.7% (6/9), while Zhang et al. (11) reported that the bypass thrombotic rate of re-Rex patients was 50% (4/8). In our research, the thrombotic rate after re-Rex shunt was 6.7% (1/15), which was significantly lower than that reported in the aforementioned studies (11, 12). The patency of bypass vessels after Rex surgery is influenced by many factors, such as the patient's age at the time of surgery, the surgical technique, selection of bypass vessels, application of postoperative anticoagulation, etc. In the Baveno VI Consensus (1), it is suggested that the patient should weigh more than 8 kg at the time of surgery. Low body weight is associated with smaller blood vessel diameters, which can affect the success of vascular anastomosis. In this study, the minimum age at first surgery was 1 year, and all the patients were at least 8 kg. Therefore, the weight and vessel size of patients in this group at the time of initial and redo surgery were not considered as the main factors affecting the success of surgery. Compared with the previous two reports (11, 12), we found that the major difference in our research is the selection of the bypass graft. Of the 15 re-Rex patients in our study, IJVs were still available to be used as bypass vessels in 11 patients (73%, 11/15). However, in Bhat et al.'s study (12), the IJVs were selected as bypass grafts in most children (86%, 56/65) in the initial Rex shunt surgery, and the remaining patients used other alternative materials as the bypass grafts. Therefore, the chances of using IJV as the bypass graft for re-Rex shunts are relatively small. Similarly, intra-abdominal vessels were all used in the previous and redo operations reported by Zhang et al. (11). As such, lower quality of intra-abdominal vessels in the reoperative procedure as well as limitations of selecting bypass grafts may be the leading causes of reduced success rate in re-Rex shunts.

To some extent, the selection of the bypass graft itself affects the patency rate of the Rex shunt. IJV was first introduced as and has been the classical bypass graft in Rex surgery. However, considering the additional surgical trauma caused by IJV interception and potential complications of losing one side of the IJV, some scholars have attempted to find alternative bypass vessels for Rex shunt surgery, especially the intrabdominal vessels. The reported patency rate of these modified bypass grafts has varied widely in the literature. Jinshan Zhang et al. (13) performed modified Rex surgery using the inferior mesenteric, ileal, or jejunal vein as the interposition graft and obtained an overall patency rate of 88.5%. In Bhat et al.'s case group (12), 86% (56/65) of the patients had the IJV used as the bypass graft, while 14% (9/65) of them had other vessels that were utilized (coronary vein, inferior mesenteric vein, splenic vein and artificial vessel), reaching patency rates of 89% and 67%, respectively. However, there is currently insufficient evidence regarding the effect of vascular selection on the success rate of Rex surgery. Therefore, the Baveno 6 consensus does not provide clear criteria for vascular selection and explains that the sources for the bypass graft vary according to the surgeon's preference, although the best results have been achieved using an internal jugular vein autograft (1).

A small diameter of the bypass conduit may predispose patients to graft thrombosis. In our research, the overall patency rate of re-Rex shunts was 93.3% (14/15). The bypass thrombosis after re-Rex shunt occurred in Patient #15, for whom the bypass was made by joining the IMV with the dilated CV with a diameter of 3 mm–4 mm. Thrombosis and surgical failure were found during the 1-year follow-up. Therefore, to achieve superior surgical outcomes, a criterion of more than 5 mm in diameter and sufficiency in length was recommended for the intra-abdominal vessels to be selected as bypass grafts (13). Bypass graft selection is the primary challenge of repeat Rex shunt surgery. When there is no access to the jugular vein, unsatisfactory intraperitoneal vessels should not be selected for the bypass graft because the risk of repeat Rex shunt surgery failure increases. Cryopreserved vessels or artificial vessels may have a sufficient diameter and length, but the high risk of thrombosis limits its application (1, 14). Therefore, we agree with the viewpoint that a Warren procedure may be a better choice if no satisfactory bypass graft could be found in a patient (11, 12).

In our study, patients with patent bypasses after re-Rex shunts achieved ideal surgical results. No rebleeding occurred in patients with patent bypass grafts after re-Rex shunt, except for patient 9 with erosive gastritis. The size of their spleens decreased substantially after the operation, and most were surprisingly not palpable at the left costal margin at the one-year follow-up visit. Concomitantly, platelet counts increased significantly, with 10 patients returning to normal levels. Furthermore, esophageal varices were alleviated significantly, and the varices were reduced to grade 0–1 levels in 9 patients. These results indicate that re-Rex surgery is able to achieve comparable outcomes as primary Rex surgery. Compared with Warren surgery, re-Rex surgery showed better relief of varices and a higher rate of platelet increase, but due to the small sample size, only a significant difference was found in the elevation of platelet counts.

Anastomotic stenosis is an important factor affecting the outcome of Rex surgery and may even eventually lead to vascular thrombosis. Stenosis was found in 3 patients in this redo Rex shunt group (20%, 3/15), which was slightly higher than previously reported in the literature after a primary Rex shunt procedure (17%) (15). Among the 3 patients with postoperative stenosis in this group, 1 patient presented with postoperative gastrointestinal bleeding (patient 1). All of them received interventional vascular dilation. After dilation treatment, the diameter of the anastomotic stenosis was enlarged in all 3 patients, and the bleeding symptom of patient 1 completely disappeared.

For Rex shunts, a previous operation may have certain influences on the postoperative effect (16). In addition to previous Rex surgery, some patients had other portal hypertension procedures, including total splenectomy, partial splenectomy, Warren procedure, and splenic artery ligation. For partial splenectomy or Warren shunt, splenic retraction and relief of hypersplenism may be influenced by poor splenic venous return to some extent. This can also explain why Patient 9 presented with erosive gastritis and intermittent gastrointestinal bleeding after the re-Rex shunt, although the bypass graft remained patent during the follow-up period. This patient finally underwent splenectomy after ineffective conservative treatments and did not develop recurrent bleeding afterward. Additionally, splenic artery ligation may also have an adverse effect on the Warren procedure. In case 6, with the Warren procedure as the redo operation, splenic artery ligation was performed at the time of the initial Rex operation. The splenic vein and the splenic venous blood flow were found to be small during the operation, and thrombosis occurred one year after the operation due to the small flow of the splenic vein. Therefore, it is necessary to fully consider the influence of previous operations and formulate a reasonable reoperational plan to achieve good surgical results.

Preoperative WHVP can be used to judge either the patency of the Rex recessus or the development of the intrahepatic portal venous system (17). Although inexperienced physicians may give false negative diagnoses, retrograde angiography can be a reliable method for skilled practitioners. In this study, all the results of retrograde portal vein angiography were confirmed by identical intraoperative exploration findings, and the coincidence rate was 100%. Moreover, all patients with well-developed intrahepatic portal veins shown by WHVP finally successfully underwent anastomosis for the re-Rex shunt procedure. However, in some cases, because of severe adjacent adhesions or heteromorphosis, the Rex recessus could be difficult to find during surgical exploration. Thus, a positive WHVP result could strongly indicate the feasibility of the re-Rex shunt and may heighten the surgeon's confidence to search carefully. Therefore, we propose that preoperative angiography should be strongly recommended for patients who need a redo operation after a failed Rex shunt.

## 5. Conclusions

Based on our results, we believe that redo-rex shunts can be finished in most patients (75%) with previous failed Rex shunts. The surgical success rate can reach as high as 90%. The bypass selection is the key point for a successful redo rex shunt. Therefore, re-Rex should be the first choice for redo operations after Rex surgery failure whenever a suitable bypass graft is available. Interventional angiography is reliable for visualizing the development of the intrahepatic portal vein system, is helpful for designing surgical plans and should be performed preoperatively. For patients with negative angiography, Warren's operation or conservative methods should be considered.

## Data Availability

The original contributions presented in the study are included in the article, further inquiries can be directed to the corresponding author.
